# RETRACTED: Waheed et al. Nitric Oxide Donor DETA/NO Inhibits the Growth of Endometrial Cancer Cells by Upregulating the Expression of RASSF1 and CDKN1A. *Molecules* 2019, *24*, 3722

**DOI:** 10.3390/molecules30224362

**Published:** 2025-11-11

**Authors:** Sana Waheed, Robert YS Cheng, Yovanni Casablanca, G. Larry Maxwell, David A Wink, Viqar Syed

**Affiliations:** 1 Department of Obstetrics & Gynecology, Uniformed Services University, 4301 Jones Bridge Road, Bethesda, MD 20814, USA; waheed1@umbc.edu (S.W.); yovanni.casablanca.mil@mail.mil (Y.C.); 2 Molecular Mechanism Section, Cancer and Inflammation Program, Center for Cancer Research, National Cancer Institute at Frederick, Frederick, MD 21702, USA; robert.cheng2@nih.gov (R.Y.C.); wink@mail.nih.gov (D.A.W.); 3 Gynecologic Cancer Center of Excellence, Department of Obstetrics and Gynecology, Uniformed Services University of the Health Sciences and Walter Reed National Military Medical Center, 8901 Wisconsin Avenue, Bethesda, MD 20889, USA; george.maxwell@inova.org; 4 John P. Murtha Cancer Center, Uniformed Services University of the Health Sciences and Walter Reed National Military Medical Center, 8901 Wisconsin Avenue, Bethesda, MD 20889, USA; 5 Department of Obstetrics & Gynecology, Inova Fairfax Hospital, 3300 Gallows Road, Falls Church, VA 22042, USA; 6 Department of Molecular and Cell Biology, Uniformed Services University, 4301 Jones Bridge Road, Bethesda, MD 20814, USA

The Journal retracts the article titled “Nitric Oxide Donor DETA/NO Inhibits the Growth of Endometrial Cancer Cells by Upregulating the Expression of RASSF1 and CDKN1A” [1], cited above.

Following publication, concerns were brought to the attention of the Editorial Office regarding the integrity of an image presented in this publication [1].

Adhering to our standard procedure, an investigation was conducted by the Editorial Office, Editorial Board, and the Uniformed Services University that identified indications of inappropriate editing and duplication between Figure 7C (AN3CA RASSF1) in this article [1] and Figure 4E (Nrf2) published in [2] by different authors, presented different experimental conditions. While the authors cooperated during the investigation, they were unable to satisfactorily explain the duplication, nor were they able to provide verifiable raw data for Editorial Board evaluation. Consequently, the Editorial Board has lost confidence in the reliability of the findings and has decided to retract this publication, as per MDPI’s retraction policy (https://www.mdpi.com/ethics#_bookmark30).

This retraction was approved by the Editor-in-Chief of the journal *Molecules*.

The author, Dr. G. Larry Maxwell, agreed, while the remaining authors did not provide a comment on this decision.
